# Essential right heart physiology for the perioperative practitioner POQI IX: current perspectives on the right heart in the perioperative period

**DOI:** 10.1186/s13741-024-00378-8

**Published:** 2024-04-09

**Authors:** Matthew D. McEvoy, Paul M. Heerdt, Vicki Morton, Raquel R. Bartz, Timothy E. Miller, POQI IX Collaborators, Matthew D. McEvoy, Paul M. Heerdt, Vicki Morton, Raquel R. Bartz, Timothy E. Miller, Stephanie Ibekwe, Jean Deschamps, Michael Grocott, Yafen Liang, Tjorvi Perry, Andrew Shaw, Rakesh Arora, Jessica Brown, Mike Tong, Subha Chatterjee, T. J. Gan, Gurmeet Singh

**Affiliations:** 1 1301 Medical Center Drive, Hi-RiSE Preoperative Optimization Clinic, Vanderbilt University Medical Center, TVC 4619, Nashville, TN 37232 USA; 2 Department of Anesthesiology, Yale School of Medicine, New Haven, USA; 3 Providence Anesthesiology Associates, Charlotte, USA; 4 Harvard Medical School, Boston, USA; 5 Department of Anesthesia, Perioperative, and Pain Medicine, Brigham and Women’s Hospital, Boston, USA; 6 Department of Anesthesiology, Duke University School of Medicine, Durham, USA

**Keywords:** Right heart, Right ventricle, Failure, Pulmonary, Physiology, Perioperative

## Abstract

As patients continue to live longer from diseases that predispose them to right ventricular (RV) dysfunction or failure, many more patients will require surgery for acute or chronic health issues. Because RV dysfunction results in significant perioperative morbidity if not adequately assessed or managed, understanding appropriate assessment and treatments is important in preventing subsequent morbidity and mortality in the perioperative period. In light of the epidemiology of right heart disease, a working knowledge of right heart anatomy and physiology and an understanding of the implications of right-sided heart function for perioperative care are essential for perioperative practitioners. However, a significant knowledge gap exists concerning this topic. This manuscript is one part of a collection of papers from the PeriOperative Quality Initiative (POQI) IX Conference focusing on “Current Perspectives on the Right Heart in the Perioperative Period.” This review aims to provide perioperative clinicians with an essential understanding of right heart physiology by answering five key questions on this topic and providing an explanation of seven fundamental concepts concerning right heart physiology.

## Introduction

As patients continue to live longer from diseases that predispose them to right ventricular (RV) dysfunction or failure, many more patients will require surgery for acute or chronic health issues. Because RV dysfunction results in significant perioperative morbidity if not adequately assessed or managed, understanding appropriate assessment and treatments is important in preventing subsequent morbidity and mortality in the perioperative period. Pulmonary hypertension, one of the leading causes of RV dysfunction, affects approximately 1% of the global population and 10% of individuals > 65 years old (Taylor et al. 2007; Hoeper et al. 2016; Peacock et al. 2007).

The overall incidence of RV dysfunction in patients undergoing non-cardiac surgery is less studied across the population; however, certain patients are known to be at increased risk of having RV dysfunction or failure. Diseases include, but are not limited to, primary and secondary pulmonary hypertension, schistosomiasis, restrictive and obstructive lung disease, obstructive sleep apnea (OSA), myeloproliferative disorders, congenital heart disease, thyroid disorders, fibrosing mediastinitis, chronic thromboembolic pulmonary diseases among many others. Understanding which patients are at risk of developing RV dysfunction will help in determining who should receive further perioperative testing and which management options should be available during the perioperative period to prevent significant morbidity and mortality (Bronze et al. 1988; Memtsoudis et al. 2010).

 In light of the increasing incidence of right heart disease, a working knowledge of right heart anatomy and physiology and an understanding of the implications of right-sided heart function for perioperative care are essential for perioperative practitioners. However, a significant knowledge gap exists concerning this topic. In fact, a recent scientific statement from the American Heart Association on the evaluation and management of right-sided heart failure concluded “it is remarkable how misunderstood are some basic concepts of right-sided heart dysfunction among practicing clinicians and the impact that such misunderstanding can have on appropriate patient management.” (Konstam et al. 2018). This manuscript is one part of a collection of papers from the PeriOperative Quality Initiative (POQI) IX Conference focusing on “Current Perspectives on the Right Heart in the Perioperative Period.” This review aims to provide perioperative clinicians with an essential understanding of right heart physiology.

## Methods

Founded in 2016, POQI is a multidisciplinary non-profit (501c3) organization whose intent is to organize consensus conferences on topics of interest in the domain of perioperative medicine. The goal is to distill the literature and make clinically relevant recommendations to improve patient care. The POQI methodology, including the use of a multi-round modified Delphi technique and the GRADE system for evidence evaluation, has been described previously (Chan et al. 2020; Martin et al. 2020; Thiele et al. 2020).

The POQI-9 consensus conference took place in New Orleans, LA from December 1–3, 2022. The objective of POQI-9 was to produce consensus statements and practice recommendations concerning Perioperative Assessment and Management of the Right Ventricle. The participants in the POQI consensus meeting were recruited based on their expertise in these domains (see Appendix 1). Conference participants were divided into three work groups. This paper details the work of Group 1 entitled “Essential Right Heart Physiology for the Perioperative Practitioner.” Groups 2 and 3 focused on the assessment and management of right heart dysfunction.

## Discussion

This POQI-9 subgroup sought to develop a consensus document providing an essential understanding of right heart physiology. Our target population includes adult patients who do not have congenital cardiac disease. As such, this consensus statement does not apply to patients with congenital or repaired congenital cardiac disease. A priori we addressed the following questions:

1. Question #1: What are the fundamental concepts for understanding right ventricular (RV) anatomy and physiology, including similarities and differences from the left ventricular (LV)?
2. Question #2: What are the components that determine RV pump function?
3. Question #3: What are the systemic consequences of right heart congestion?
4. Question #4: What is the physiologic cascade that occurs with declining right ventricular performance?
5. Question #5: What are physiologic stresses on right heart performance that occur in the perioperative period?

Each section of the “Discussion” section will be introduced with summary statements concerning key concepts related to understanding the right heart followed by a narrative review of the latest evidence.

### Right heart anatomy

#### Concept #1a

The right ventricle (RV) is fundamentally different in anatomy and physiology from the left ventricle (LV).

#### Concept #1b

Changes in coronary blood flow in the setting of pulmonary hypertension make the RV more susceptible to ischemia from systemic hypotension.

Increased recognition of the right ventricular (RV) contribution to overall cardiovascular performance in both health and disease has prompted the publication of several monographs and focused reviews (Naeije 2015; Gittenberger-de Groot et al. 2015; Edward et al. 2023; Vandenheuvel et al. 2013; Sanz et al. 2019; Dell'Italia 2012; Walker and Buttrick 2009; Haddad et al. 2008). In addition, professional organizations have issued statements highlighting knowledge gaps and underscoring the need for better methods to assess function along the course of RV adaptation from dysfunction to failure (Konstam et al. 2018; Lahm et al. 2018; Voelkel et al. 2006). Within this context, a scientific statement from the American Heart Association on the perioperative management of patients with pulmonary hypertension was recently published (Rajagopal et al. 2003).

While the normal RV is generally characterized as a thin-walled structure largely wrapped around the interventricular septum that ejects blood at low pressure into the pulmonary circulation, the fetal RV functions at high pressures and provides the majority of systemic blood flow. As such, the RV does not begin to assume its eventual structure and shape until pulmonary vascular resistance markedly falls after birth when the lungs expand, and the ductus arteriosis and foramen ovale close (Sanz et al. 2019).

The RV is regarded as having three regions (inflow, apical, and outflow) arranged in a “boot-like” or triangular configuration along the septum (Walker and Buttrick 2009). In the free wall, superficial circumferential fibers predominate and wrap around the LV with a subendocardial layer of longitudinal fibers passing from the apex to the tricuspid annulus and outflow tract (Sanz et al. 2019). The midline is formed by the interventricular septum comprised of oblique helical fibers that cross each other at 60° angles similar to the LV-free wall (Buckberg and Hoffman 2014). Fiber orientation and distribution influence the function of both ventricles with transverse fibers producing circumferential strain and helical fibers causing longitudinal strain when oblique fibers at reciprocal angles thicken and coil. Overall, the predominant strain in terms of work is longitudinal (Haddad et al. 2008). For the RV, basilar wrap-around circumferential fibers and the septum primarily dictate systolic function (Buckberg and Hoffman 2014).

Internally, the inflow tract and apical regions include papillary muscles and more coarse trabeculation than the LV and transition into the non-trabeculated outflow tract below the pulmonic valve (Walker and Buttrick 2009). Although increasingly sophisticated molecular biology techniques have highlighted the complexity of cardiac morphogenesis and the origin of the primitive cardiac tube, it is clear that differences in LV and RV structure and function reflect variant embryology. For the RV, different areas are conventionally regarded as developing from different primitive cardiac tube components with the ventricular portion giving rise to the inflow and apical regions (as well as the LV), and the outflow tract arising from the bulbous chordis (Dell'Italia 2012). Particular interest has been focused on the development of the outflow tract given its role in congenital heart disease and as a major site for arrhythmogenic cardiomyopathy (Boukens et al. 2016). In addition, substantial pressure gradients between the RV and pulmonary artery have been reported with sympathetic stimulation or rapid afterload reduction due to a hypercontractile outflow tract (Raymond et al. 2019; Kroshus et al. 1995). Some authors have suggested that outflow tract narrowing early in systole is an adaptive response that protects the pulmonary circulation from high pressure and ejection velocity (March et al. 1962). However, the synchrony of inflow-to-outflow shortening is also affected by the depressive effects of anesthetics and autonomic blockade (Heerdt and Pleimann 1996).

The majority of blood supply to the RV free comes from the right coronary artery (RCA) with branches perfusing the atrioventricular (AV) and sinoatrial (SA) nodes. In most patients, the RCA is the predominant source of flow to the posterior descending artery perfusing the inferior LV wall and posterior third of the interventricular septum. The remaining two-thirds of the interventricular septum is supplied by the left anterior descending coronary artery which may also perfuse some of the medial RV-free walls (Ikuta et al. 1988). It is well appreciated that some patients have a supernumerary coronary vessel termed the conus artery that arises from an ostium behind the right cusp of the aortic valve that is either distinct from or close to the RCA ostium and courses over the antero-superior surface of the RV before terminating near the anterior interventricular groove (Schlesinger et al. 1949). The conus artery has a lower incidence of occlusion than the RCA or LCA and can provide collateral flow to these vessels, and may contribute to the preservation of RV outflow tract function in the setting of acute RV infarction (Dell'Italia 2012). Venous drainage of the RV differs from the LV in that most flow bypasses the coronary sinus and empties directly into the right heart (Sirajuddin et al. 2020). Anatomically, venous drainage occurs via small Thebesian vessels, along with the right marginal vein, a series of anterior cardiac veins, and the infundibular veins. In roughly a quarter of the population, a small cardiac vein enters the coronary sinus at a point close to the coronary sinus/RA junction. Table 1 provide a comparison of major anatomical components of the RV and LV.

**Table 1:** Comparative characteristics of normal left (LV) and right (RV) ventricles^a^

Feature	LV	RV
Structure (Sanz et al. 2019; Dell'Italia 2012)	Elliptical	Triangular, tripartite (inflow, apical, outflow)
Wall thickness (mm) (Haddad et al. 2008)	7–11	2–5
Mass (g/m^2^) (Haddad et al. 2008)	87 ± 12	26 ± 5
End-diastolic volume (mL) (Haddad et al. 2008)	66 ± 12	75 ± 13
Ejection fraction (%) (Haddad et al. 2008)	67 ± 5	61 ± 7
Stroke work index (g/m^2^/beat) (Haddad et al. 2008)	50 ± 20	8 ± 2
Outflow resistance (dyne-sec-cm-5) (Haddad et al. 2008)	1100	70
Pressure–volume relationship (Heerdt and Dickstein 1997)	Rectangular	Triangular to rectangular
Optimal adaptive capacity (Edward et al. 2023; Vandenheuvel et al. 2013)	Pressure load	Volume load
Coronary perfusion interval (Crystal and Pagel 2018)	Diastole	Systole and diastole
Ischemic tolerance (Crystal and Pagel 2018)	Relatively poor	Relatively good

^a^Reference numbers shown in parentheses

The dynamics of coronary perfusion vary substantially between the RV and LV. In a recent extensive review, Crystal and Pagel described the distinctive characteristics of RV perfusion which promote a relative resistance to myocardial ischemia and dysfunction, and how this protection may become compromised in patients with acute pulmonary hypertension (Crystal and Pagel 2018). These factors are primarily related to the lower developed intracavitary and tissue pressures during systole in the normal RV and are as follows: (1) in contrast to the LV, blood flow throughout the entire cardiac cycle; (2) lower baseline oxygen uptake and the ability to at least partially compensate for reduced blood flow by increasing oxygen extraction; (3) preservation of energy stores during decreased perfusion by downregulation of oxygen demand; (4) while epicardial coronary stenosis disproportionally impairs perfusion of LV subendocardium, reduced perfusion in the RV is transmurally uniform; (5) potentially retrograde perfusion from the RV cavity through the Thebesian veins and extensive collateral connections.

**Fig. 1:**
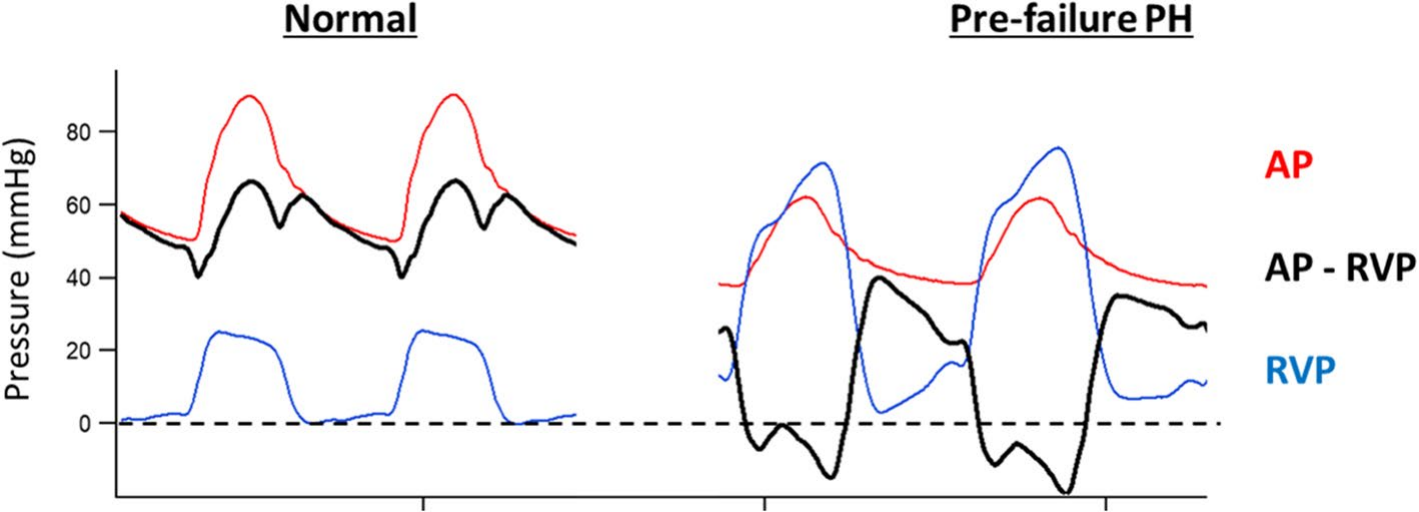
Comparison of pressure in the ascending aorta (AP, in red) and right ventricle (RVP, in blue) along with the pressure gradient between them (AP-RVP, in black) driving coronary perfusion. Under normal conditions (left panel), AP > RVP at all times facilitates RV perfusion in both systole and diastole. In contrast, in the setting of marked pulmonary hypertension (PH) (right panel), RVP can exceed AP during the systolic portion of the cardiac cycle thus eliminating the positive pressure gradient during systole and limiting perfusion to the diastolic interval. Data were obtained during an experimental study of progressive pulmonary embolization under a protocol approved by the institutional animal care and use committee. The figure is reproduced with permission from the PeriOperative Quality Initiative (POQI)

Differences in myocardial perfusion during systole can be of particular concern in the perioperative setting. As shown in Fig. 1, the low RV pressure normally generated during systole permits coronary arterial flow during both systole and diastole due to a continuous aortic root-RV myocardial pressure gradient. However, with afterload stress, the increased RV systolic pressure necessary to maintain ejection will increase oxygen demand and if combined with systemic hypotension can result in decreased RV perfusion and supply/demand mismatch. Not surprisingly, in the setting of pulmonary hypertension, impaired RV systolic function secondary to ischemia can become quickly apparent when acute systemic hypotension is superimposed and the systolic component of perfusion is lost (Steppan and Heerdt 2021).

Electrical activation of the RV-free wall spreads from the AV node via branches of the right bundle of the His-Purkinje system (Padala et al. 2021) and is generally coincident with that of the LV although septal contraction may precede that of the RV-free wall. Within the RV, contraction is typically heterogenous with inflow tract contraction preceding that of the outflow tract by 30 to 60 ms, most likely reflecting at least in part regional differences in the conducting apparatus (Heerdt and Dickstein 1997).

### Right heart physiology

#### Concept #2a

In contrast to the LV, normal RV pump function is more sensitive to changes in afterload and more tolerant of changes in preload.

#### Concept #2b

LV contraction is important for normal RV function and a significant percentage of RV outflow is generated by LV contraction.

### Physiology

Despite structural and functional differences, the performance of both the LV and RV as volume pumps is largely dictated by the same factors (preload, afterload, and contractility). That said, specific features of each of these factors as well as their regulation vary between chambers. In relation to these components Table 2 summarizes the pharmacology and physiology by receptor sites in the right heart.

**Table 2:** Receptor pharmacology and physiology affecting the right heart

Medication	Receptor site action	Clinical relevance
Phenylephrine (10 mcg/min–200 mcg/min)	Pure α_1_ receptor agonist	Increases systemic vascular resistance (SVR), potential to increase pulmonary vascular resistance
Norepinephrine (0.02 mcg/kg/min–0.3 mcg/kg/min Or (1–20 mcg/min)	α_1_, β_1_ receptor agonist	Increases in SVR may have some effect on contractility and HR
Epinephrine (0.02 mcg/kg/min–0.3 mcg/kg/min) Or (1–20 mcg/min)	α_1_, β_1_, β_2_ receptor agonist	Increases SVR, increases HR, increases contractility
Dobutamine (0.5 mcg/kg/min–20 mcg/kg/min)	β_1_, β_2_ receptor agonist	Increases HR, increases contractility, may lead to hypotension in some patients
Dopamine (0.5 mcg/kg/min–10 mcg/kg/min)	δ_1_, α_1_, β_1_, β_2_ agonist	Increases SVR, Increased HR, Increased contractility
Isoproterenol (2–10 mcg/min)	β_1_, β_2_ agonist	Increases HR, increases contractility
Milrinone (0.1 mcg/kg/min–0.5 mcg/kg/min)	Phosphodiesterase III inhibitor	Increases contractility and decreases pulmonary vascular resistance (PVR), may lead to hypotension
Vasopressin (0.02 U/min–0.04 U/min)	V_1_ receptor agonist	Increases SVR through splanchnic vessels, no effect on pulmonary vasculature can counteract Milrinone-induced decreases in SVR, no effect on HR
Inhaled Nitric Oxide (1–20 PPM)	Activates soluble guanylate cyclase	Decreases PVR
Inhaled Epoprostenol (0.01–0.1 mcg/kg/min)	Synthetic prostacyclin	Decreases PVR

#### Preload

In that sarcomere length at the end of diastole is indicative of myocardial preload, ventricular compliance determined by the end-diastolic pressure/volume relationship plays a major role. For the LV, diastolic compliance is largely determined by the inherent viscoelastic properties of the thick wall and is normally independent of the RV. In contrast, for the thin-walled, highly distensible RV, the pericardium, intrathoracic pressure, and LV influence diastolic compliance (Sanz et al. 2019). In the progression of RV adaption to dysfunction with pulmonary hypertension, the influence of pericardial restraint on diastolic compliance may initially be reduced as the RV hypertrophies. However, restrictions in diastolic compliance become increasingly important as the disease progresses and ventricular dilation with wall thinning occurs.

#### Afterload

Conceptually, ventricular afterload is the end-systolic wall tension that results from the opposition to sarcomere shortening and ejection of blood. The forces opposing ejection can be broadly characterized as resistive, elastic (compliant), and reflective (coming back toward the heart late in systole) and vary over the course of ejection. This distinction has particular functional significance for RV for several reasons. First, although RV afterload is commonly expressed as steady-state (non-pulsatile) pulmonary vascular resistance (mean pressure/mean flow), 30–50% of the work performed by the chamber is pulsatile, i.e., goes toward overcoming the elastic and reflective forces (Grandin et al. 2017). Second, in comparison to the LV, acute increases in RV afterload have a much greater impact on pump function. In this context, acute insults such as pulmonary embolism can have profound effects. When the load stress is chronic, however, the RV does have the ability to adapt to both heterometric and homeometric processes (Edward et al. 2023). Finally, in the perioperative and critical care environments, interventions such as mechanical ventilation and positive end-expiratory pressure can increase both non-pulsatile and pulsatile determinants of afterload. As such, the need for a better understanding of RV afterload and the definition of more complete metrics to quantify afterload have been identified as a research priority (Lahm et al. 2018).

#### Contractility

Despite differences in myocyte size (RV are ~ 15% smaller than those from the LV) and the suggestion of differences in sarcomere shortening and intracellular calcium transients (Walker and Buttrick 2009; Erickson and Tucker 1986), the ability of LV and RV myocytes to perform work over a range of loading conditions is similar. However, consistent with structural and geometric differences between the chambers, in the intact heart the RV work/load relationship is substantially different from that of the LV. Traditionally, RV contraction has been characterized as having four phases: (1) a “bellows effect” produced by inward movement of the RV free wall; (2) longitudinal shortening pulling the tricuspid annulus toward the apex; (3) late contraction of the RV outflow tract; and (4) LV augmentation of RV contraction via contiguous circumferential fibers and septal shortening. Enhanced experimental and imaging techniques have expanded our understanding of how transverse and helical muscle fibers within the RV-free wall and septum interact in a sequential fashion to produce force and eject blood. In particular, the data indicate that longitudinal shortening results primarily from coiling of helical fibers not contraction of longitudinal muscle layers, and that the septum plays a major role in generating longitudinal strain (Buckberg and Hoffman 2014). These concepts underscore the importance of considering ventricular interdependence since a substantial portion of RV systolic function is ultimately provided by LV contraction and septal movement. In an intricate study involving electrical isolation of the RV and LV, Damiano et al. demonstrated that if LV contraction is maintained while RV-free wall movement is prevented, when RV filling is optimized more than 60% of the beating RV pressure and 80% of the pulmonary arterial flow are produced (Damiano et al. 1991), highlighting the contribution of LV and septal contraction to RV function. Subsequent studies have focused on this phenomenon as it relates to the impact of LV mechanical assist devices on RV function. When RV pressure and volume become markedly increased or critical areas of the septum are infarcted, interdependence can transition to “ventricular interference” as a leftward shift in the interventricular septum impedes LV filling, or loss of septal helical motion impairs RV longitudinal shortening.

**Fig. 2:**
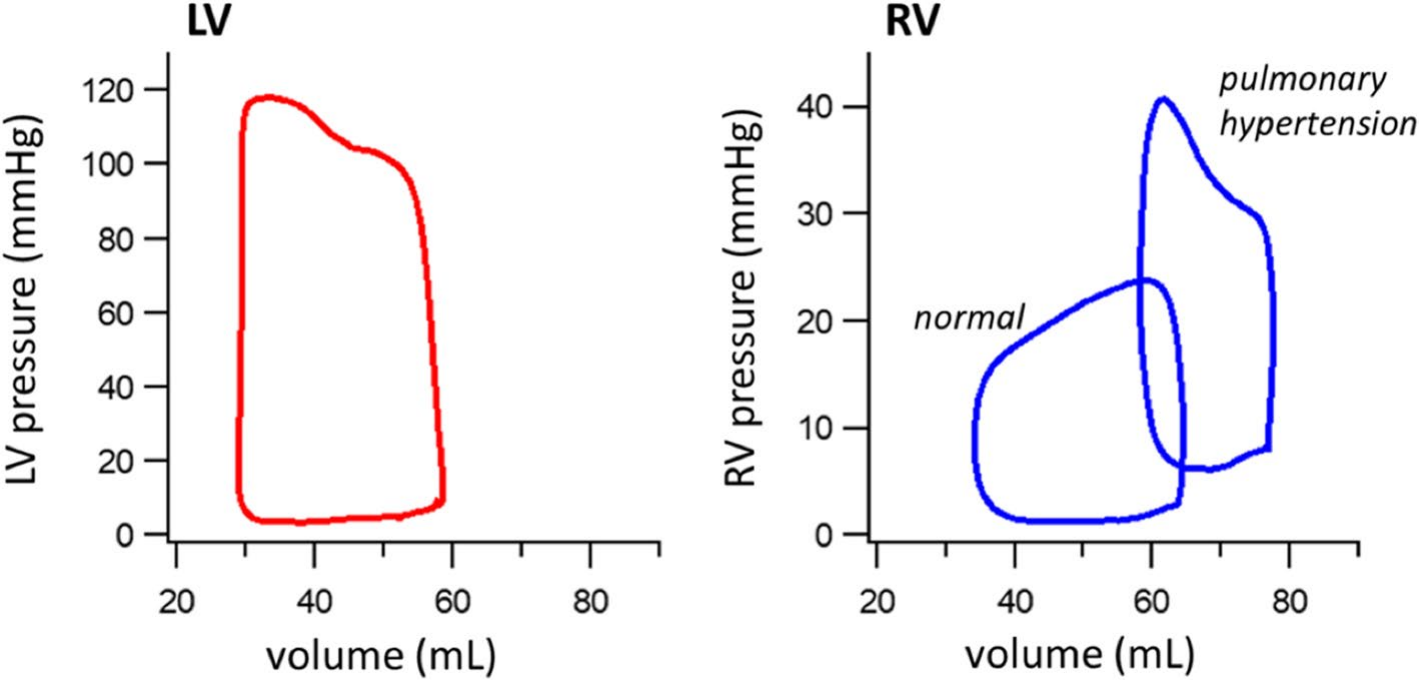
Example of left (LV) and right (RV) ventricular pressure–volume loops (animal model). LV loops are normally rectangular with a well-defined upper left corner corresponding to end-systole, which occurs shortly after maximal pressure is reached. In contrast, under normal, low-pressure conditions the RV loop is more triangular with a less well-defined upper left corner that occurs well after maximal pressure is reached. However, in the setting of pulmonary hypertension, the RV loop transitions to a morphology more similar to a normal LV pressure–volume loop. Data were obtained during an experimental study of progressive pulmonary vasoconstriction under a protocol approved by the institutional animal care and use committee. The figure is reproduced with permission from the PeriOperative Quality Initiative (POQI)

Ultimately, the interaction of preload (both the magnitude of end-diastolic volume and the associated pressure) with contractility and afterload (both the magnitude and timing of peak load) dictate characteristics of the RV pressure–volume relationship (Fig. 2). Under normal low pressure, low afterload conditions the timing of peak pressure in the RV occurs earlier in the cardiac cycle than in the LV and this difference is reflected in the shape of the pressure–volume loop. However, with increased afterload the timing of peak RV pressure can shift to late systole causing the RV pressure–volume loop to more closely resemble that of the LV.

### Right heart dysfunction: venous congestion and physiologic consequences

#### Concept #3

Venous congestion is a consequence of right heart failure and may contribute to inadequate perfusion and organ dysfunction.

It is common for clinicians to consider the effect of left heart failure, especially poor cardiac output, on system organ dysfunction. However, the effects of right heart failure on organ dysfunction are often not taken into account. While the left heart produces the inlet pressure (i.e., mean arterial pressure) that promotes organ perfusion, right heart failure can profoundly increase the outlet pressure from an organ (i.e., venous pressure and central venous pressure), thereby reducing the perfusion pressure even in the setting of normal arterial pressure. Right heart failure impairs the forward flow of deoxygenated blood causing elevated venous pressure, the hallmark sign of right heart failure. This leads to a pathological milieu of peripheral and visceral venous congestion. Peripheral venous congestion will lead to jugular venous distension (JVD), a classic sign of venous hypertension, and lower extremity edema. As the right heart failure progresses, patients will experience increased exercise intolerance and chronic fatigue (Konstam et al. 2018). In hospitalized patients, JVD due to right heart failure is associated with an increased risk of adverse events, 30-day mortality, and 1-year all-cause mortality (Chernomordik et al. 2016).

Beyond peripheral venous congestion, it has been shown that visceral venous congestion due to RV dysfunction correlates with impaired liver, kidney, and intestinal function, and cardiac cachexia (Valentova et al. 2013). Heart failure leading to kidney failure has been termed cardiorenal syndrome. In decompensated right heart failure with reduced ejection fraction (HFrEF), chronic elevation of central venous pressure and decreased cardiac output lead to the activation of vasopressin, renin–angiotensin–aldosterone system (RAAS), and the sympathetic nervous system resulting in vasoconstriction with sodium and water retention. This leads to decreased renal perfusion, ischemia of the kidney, and decreased glomerular filtration rate creating a clinical picture of decreased urine output and increased fluid retention (Konstam et al. 2018). Similarly, cardiohepatic syndrome, or congestive hepatopathy, is a result of hepatic congestion and reduced perfusion to the liver. In chronic right heart failure (RHF), symptoms of liver involvement can be vague early on, often mimicking symptoms of cholelithiasis such as right upper quadrant pain and nausea (Samsky et al. 2013). As RHF progresses, symptomatology progresses as hepatic venous pressures continue to rise, thereby decreasing hepatic oxygen delivery (Samsky et al. 2013). As the syndrome persists, cardiac cirrhosis is a likely end result (Konstam et al. 2018). Chronically increased CVP and reduced CO can also lead to impaired gastrointestinal function as a result of visceral congestion. The intestine is typically well-perfused by the splanchnic circulation. However, in the presence of venous congestion activating the sympathetic nervous system and subsequent constriction of blood vessels and perfusion reduction, intestinal ischemia and inflammation occur (Konstam et al. 2018). The consequences of these changes in the gastrointestinal tract lead to the reduction of nutrient absorption, anemia, hypoalbuminemia, and cachexia (Konstam et al. 2018). Due to the combination of cardiorenal interactions, hepatomegaly, and reduced gastrointestinal function, cardiac cachexia is a common result. Independent of age or functional class, cardiac cachexia is predictive of increased mortality in patients with heart failure (Cicoira et al. 2007). Cachexia further worsens the inflammatory response and its consequences such as cardiac and skeletal muscle changes, worsening cardiac function, and reducing physical activity tolerance. This creates a vicious cycle of loss of muscle mass, which only potentiates the cachectic process (Cicoira et al. 2007). Taken together, venous congestion as a consequence of worsening right heart failure leads to reduced organ perfusion that results in significant end-organ dysfunction.

#### Concept #4

Predictable physiologic disturbances occur in the progression from normal right heart function to right heart failure.

Predictable changes occur in right heart failure (RHF). Since the right heart is a lower-pressure system, it is more sensitive to alterations in afterload. Due to ventricular interdependence, any modest change in pulmonary vascular resistance, such as in the presence of pulmonary hypertension, will create an increase in RV afterload causing the RV stroke volume to subsequently decrease, and compromise left ventricular filling due to right to left septal shifting (Rosenkranz et al. 2020). This interaction leaves the LV underfilled due to the RV congestion, yet left-sided pressures are elevated. The result is a decrease in cardiac output. This becomes particularly challenging during scenarios that cause increased venous return and additional increases in RV volume, such as during times of activity.

As the RV volumes continue to increase, functional tricuspid regurgitation will be the result causing worsening RV dilation and subsequent decrease in left ventricular filling and decreased left ejection fraction. Due to the right ventricle failing to operate as a forward pump, the systemic venous circulation becomes impaired resulting in systemic venous congestion which causes jugular venous distention, lower extremity edema, hepatosplanchnic congestion, and gut edema (Wenger et al. 2017). Due to increased left heart pressures, we expect to see dyspnea and increased fatigability associated with congestive heart failure. An increase in right-sided filling pressures also causes the coronary blood flow to become compromised due to the right ventricle dilation and hypertrophy. The compromised flow then creates additional oxygen demand which normal coronary flow is unable to satisfy (Rajagopal et al. 2023).

In the presence of pulmonary artery hypertension (PAH) due to left ventricular failure, the RV afterload gradually increases (Konstam et al. 2018). The chronicity of PAH and RHF will render the RV much less tolerant to volume overload, promoting a compensated right heart failure into a decompensated state due to ventricular remodeling, ultimately leading to fibrosis of the right ventricle. Once this occurs, the expected increase in pulmonary vascular resistance and right atrial pressures is coupled with a decreased cardiac output and pulmonary arterial pressure, potentially leading to cardiogenic shock and death (Rajagopal et al. 2023).

### Modifiable perioperative stress

#### Concept #5

Predictable, modifiable physiologic stresses that occur in the perioperative period include surgical (hypovolemia, pneumoperitoneum), physiologic (hypoxia, hypercarbia, and hypotension), and anesthetic (positive pressure ventilation) factors.

The perioperative period is known to create physiologic stress of varying degrees that are of particular importance to right heart physiology. These stressors are predictable and frequently modifiable and fall into three main categories: surgical, anesthetic, and physiologic. Table 3 provides a list of common, predictable stressors, the stress response on the RV, and systemic hemodynamics, and an example of how this may be encountered in the perioperative period. The list is to serve as a guide for consideration but not an exhaustive detailing of potential perioperative stressors.

**Table 3:** Perioperative stressors and right heart physiologic responses

Stressor	Stress response	Examples
Systemic hypotension	↓Systolic and diastolic pressure leading to reducing coronary perfusion, especially with elevated RV pressure	Induction; rapid blood loss
Hypoxemia	↑Pulmonary vascular resistance	Single lung ventilation; reduced minute ventilation in MAC cases; postoperative opioid-related respiratory depression
Hypercarbia	↑Pulmonary vascular resistance	Pneumoperitoneum; release of tourniquet
Acidosis	↑Pulmonary vascular resistance, ↓systemic BP, and reduced response to vasopressors	Shock with increased lactate, hypercapnia, ketoacidosis
Positive pressure ventilation	↑Pulmonary vascular resistance	Intubation; high inspired pressures that create more dead space; high PEEP
Hypervolemia	Elevated PCWP can increase PA, RV, and RA pressures and if acute can reduce RV output or cause acute TR	Excessive IVF administration; TACO; steep Trendelenburg
Hypovolemia	Low filling pressures can greatly reduce RV output	Rapid acute blood loss; steep reverse Trendelenburg especially with pneumoperitoneum; prone position with increased chest pressure or abdominal pressure retarding IVC flow for RA/RV filling

## Conclusions

The goal of this narrative review was to provide the perioperative practitioner with an essential understanding of the right heart physiology. Several key points should be mastered for clinical application. First, the RV is fundamentally different in anatomy and physiology from the LV, and changes in coronary blood flow in the setting of pulmonary hypertension make the RV more susceptible to ischemia from systemic hypotension. Second, in contrast to the LV, normal RV pump function is more sensitive to changes in afterload and more tolerant of changes in preload, and LV contraction is important for normal RV function as a significant percentage of RV outflow is generated by LV contraction through ventricular interdependence. Third, venous congestion is a consequence of right heart failure and is a significant contributor to inadequate perfusion and organ dysfunction. Fourth, part of the understanding of right heart function is that there are predictable physiologic disturbances that occur in the progression from normal right heart function to right heart failure. Finally, all of this finds clinical relevance for perioperative practitioners because there are predictable, modifiable physiologic stresses that occur in the perioperative period. Other papers in this series will expand upon this knowledge base to incorporate specific strategies for the assessment and management of right-heart dysfunction and failure in the perioperative period.

## Data Availability

N/A.
