# Correction: Essential right heart physiology for the perioperative practitioner POQI IX: current perspectives on the right heart in the perioperative period

**DOI:** 10.1186/s13741-025-00608-7

**Published:** 2025-11-28

**Authors:** Matthew D. McEvoy, Paul M. Heerdt, Vicki Morton, Raquel R. Bartz, Timothy E. Miller, POQI IX Collaborators, Matthew D. McEvoy, Paul M. Heerdt, Vicki Morton, Raquel R. Bartz, Timothy E. Miller, Stephanie Ibekwe, Jean Deschamps, Michael Grocott, Yafen Liang, Tjorvi Perry, Andrew Shaw, Rakesh Arora, Jessica Brown, Mike Tong, Subha Chatterjee, T. J. Gan, Gurmeet Singh

**Affiliations:** 1 1301 Medical Center Drive, Hi-RiSE Preoperative Optimization Clinic, Vanderbilt University Medical Center, TVC 4619, Nashville, TN 37232 USA; 2 Department of Anesthesiology, Yale School of Medicine, New Haven, USA; 3 Providence Anesthesiology Associates, Charlotte, USA; 4 Harvard Medical School, Boston, USA; 5 Department of Anesthesia, Perioperative, and Pain Medicine, Brigham and Women’s Hospital, Boston, USA; 6 Department of Anesthesiology, Duke University School of Medicine, Durham, USA

**Correction** **:** **Perioper Med 13, 27 (2024)**

**https://doi.org/10.1186/s13741-024-00378-8**

Following the publication of the original article (McEvoy et al. 2024), the authors identified an error in the Competing interests section. The section should read:

**Competing interests**

Mike Grocott is the Editor-in-Chief of the Perioperative Medicine.

**The old statement of the Competing interests**: The authors declare no competing interests.

The Competing interests section has been updated above and the original article (McEvoy et al. 2024) has been corrected.
